# Protective Efficacy of *Lactobacillus plantarum* Postbiotic beLP-K in a Dexamethasone-Induced Sarcopenia Model

**DOI:** 10.3390/ijms26157504

**Published:** 2025-08-03

**Authors:** Juyeong Moon, Jin-Ho Lee, Eunwoo Jeong, Harang Park, Hye-Yeong Song, Jinsu Choi, Min-ah Kim, Kwon-Il Han, Doyong Kim, Han Sung Kim, Tack-Joong Kim

**Affiliations:** 1 Division of Biological Science and Technology, Yonsei University, Wonju 26493, Republic of Korea; mjy@yonsei.ac.kr (J.M.); drlogos@naver.com (J.-H.L.); jew0108@naver.com (E.J.); phraa@naver.com (H.P.); shy9987@naver.com (H.-Y.S.); wlstnbnm@naver.com (J.C.); mina1218@yonsei.ac.kr (M.-a.K.); 2 Research & Development Center, bereum Co. Ltd., Wonju 26362, Republic of Korea; kihan@bereum.com; 3 Department of Biomedical Engineering, Yonsei University, Wonju 26493, Republic of Korea; dykim2650@naver.com (D.K.); hanskim@yonsei.ac.kr (H.S.K.); 4 Research & Development Center, Doctor TJ Co. Ltd., Wonju 26494, Republic of Korea

**Keywords:** sarcopenia, *Lactobacillus plantarum*, postbiotic, beLP-K, dexamethasone, FoxO3α, MAFbx/atrogin-1, MuRF1

## Abstract

Sarcopenia is characterized by a reduction in muscle function and skeletal muscle mass relative to that of healthy individuals. In older adults and those who are less resistant to sarcopenia, glucocorticoid secretion or accumulation during treatment exacerbates muscle protein degradation, potentially causing sarcopenia. This study assessed the preventive effects and mechanisms of heat-killed *Lactobacillus plantarum* postbiotic beLP-K (beLP-K) against dexamethasone (DEX)-induced sarcopenia in C2C12 myotubes and Sprague-Dawley rats. The administration of beLP-K did not induce cytotoxicity and mitigated cell damage caused by DEX. Furthermore, beLP-K significantly reduced the expression of forkhead box O3 α (FoxO3α), muscle atrophy f-box (MAFbx)/atrogin-1, and muscle RING-finger protein-1 (MuRF1), which are associated with muscle protein degradation. DEX induced weight loss in rats; however, in the beLP-K group, weight gain was observed. Micro-computed tomography analysis revealed that beLP-K increased muscle mass, correlating with weight and grip strength. beLP-K alleviated the DEX-induced reduction in grip strength and increased the mass of hind leg muscles. The correlation between beLP-K administration and increased muscle mass was associated with decreased expression levels of muscle degradation-related proteins such as MAFbx/atrogin-1 and MuRF1. Therefore, beLP-K may serve as a treatment for sarcopenia or as functional food material.

## 1. Introduction

With the increase in life expectancy and aging world population, sarcopenia has become a significant health problem. Sarcopenia is defined as a progressive and systemic skeletal muscle disease that induces loss of muscle mass and function [1,2]. The known causes of sarcopenia include aging, cancer, oxidative stress, mitochondrial dysfunction, malnutrition, low physical activity, inflammatory responses, sepsis, diabetes, renal failure, and Cushing’s syndrome [3,4,5,6]. Each pathological condition can induce various metabolic changes in the body, which may include an imbalance in glucocorticoid (GC) hormone levels. In such pathological states, GC imbalance resulting from metabolic disturbances is closely associated with the development of sarcopenia [7,8]. Sarcopenia is often associated with aging; previous studies have reported that skeletal muscle mass and strength gradually decline by up to 50% from the early 40s to the 80s [9]. Despite its severity, sarcopenia has only recently been classified as a severe disease. In 2010, the European Working Group on Sarcopenia began to define sarcopenia as a disease characterized by low muscle strength and muscle mass, and since then, several studies have gradually established its definition [10]. Following these research findings, in 2016, the World Health Organization (WHO) officially recognized sarcopenia by granting it the International Classification of Diseases, 10th Revision, Clinical Modification code. Similarly, South Korea officially classified sarcopenia as a disease by assigning it the code M62.5 in the Korean Standard Classification of Diseases, 8th Revision, in 2021 [11]. According to a study by the Global Burden of Disease, the number of patients with sarcopenia, which was 494 million in 2020, is predicted to increase to 1.06 billion by 2050, an increase of more than 115%, thereby raising interest in its prevention and treatment [12,13]. According to previous studies, the prevalence of sarcopenia among older adults in South Korea has been reported to range from approximately 6.8% to 13.1%, depending on the diagnostic criteria, highlighting its significance as a public health concern [14,15]. However, the definition and diagnostic criteria for sarcopenia have not yet been standardized, resulting in differences among studies. A recent study on the elderly reported that the prevalence of sarcopenia based on muscle mass was 4–22% and based on walking speed and grip strength was 4–34% and 4–16%, respectively, showing large differences depending on the assessment criteria and suggesting that future improvements in the assessment criteria are needed [16].

Muscle cell loss due to the inhibition of muscle protein synthesis and autophagy-lysosome processes are the main mechanisms underlying sarcopenia [17,18]. GCs, which activate the ubiquitin–proteasome pathway, are 21-carbon steroid hormones, which have been widely used for nearly 50 years due to their immunosuppressive and anti-inflammatory effects. However, when taken at high doses or for long periods, they can cause potential side effects such as sarcopenia, osteoporosis, glaucoma, and cataracts [19,20]. Sarcopenia is an important side effect of GCs. They activate muscle protein degradation through the activity of the ubiquitin–proteasome system (UPS), rendering muscle fibers thin and reducing muscle mass [21,22]. Dexamethasone (DEX), a synthetic GC, is widely used due to its therapeutic efficacy against allergic and inflammatory diseases. However, excessive use of GCs can reduce muscle protein synthesis and promote protein degradation, leading to weight loss and sarcopenia [23,24,25]. Muscle fibers are distributed between slow-twitch muscle fibers (STMFs) and fast-twitch muscle fibers (FTMFs), of which type I muscle fibers, which are STMFs, are only mildly affected by GCs such as DEX; however, type II muscle fibers, which are FTMFs, are selectively affected by GCs, resulting in an overall decrease in muscle mass [26]. Sarcopenia is a multifactorial disease associated with several causes, including apoptosis, mitochondrial alterations, autophagy inhibition, lysosomal system dysfunction, and the UPS. Among them, the UPS is known as a major mechanism involved in muscle protein degradation [27,28]. GC-induced UPS activation stimulates the expression of two important muscle-specific E3 ubiquitin ligases, muscle atrophy f-box (MAFbx)/atrogin-1 and muscle RING-finger protein-1 (MuRF1) [29]. The transcription and translation of genes encoding these ligases mediate the activation of proteasomes, which are directly involved in protein degradation and the ubiquitination of several muscle proteins, thereby inhibiting muscle protein synthesis and contributing to muscle loss [30,31,32,33]. RING-finger E3 ligase family proteins, including MuRF1, exist in over 600 different forms in human cells [34,35,36]. The transcription factor, forkhead box O3 α (FoxO3α), which regulates E3-ubiquitin ligase transcription, is associated with GC-induced sarcopenia. FoxO3α overexpression mediates E3-ubiquitin ligase transcription, activating MAFbx/atrogin-1 and MuRF1, thereby reducing muscle protein levels [37]. It regulates human physiological functions by inducing protein ubiquitination, thereby modifying mechanisms, regulating transcriptional and translational activities, and altering signaling pathways [38,39]. Several studies have investigated the treatment and prevention of DEX-induced sarcopenia, and many mechanisms have been identified. In addition, it has been suggested that methods such as exercise can alleviate sarcopenia, but definitive pharmacological treatment has not yet been established, emphasizing the need for continuous research and drug development [40].

As sarcopenia research aims to improve quality of life amid accelerated aging, increasing incidence of chronic diseases, advances in space technology, and increased interest in healthcare, identifying agents that can prevent or mitigate muscle loss is necessary. However, to date, drugs approved by the U.S. Food and Drug Administration for the treatment of sarcopenia have not been reported. For sarcopenia treatment, various candidate substances, such as selective androgen receptor modulators, dehydroepiandrosterone, and myostatin inhibitors, are being studied in early clinical stages, but further studies are still required to address interindividual differences in efficacy and to ensure safety [41]. In this context, studies using natural extracts such as curcumin and ginseng for sarcopenia treatment have been conducted [42,43], and recently, an increasing trend in postbiotics research has been observed. Particularly, *Lactiflantibacillus plantarum* KM-2 was reported to effectively reduced the expression levels of the E3-ubiquitin ligases, MAFbx/atrogin-1 and MuRF1, in cellular and animal models, while enhancing FoxO3α, Akt, and mTOR phosphorylation, contributing to muscle protein synthesis [44]. Furthermore, *Lactobacillus rhamnosus* IDCC3201 has been shown to alleviate DEX-induced muscle size and grip loss in animal models [45]. Although the WHO and Food and Agriculture Organization acknowledge that probiotic administration in adequate amounts is beneficial for health, safety concerns associated with the use of live microorganisms persist; thus, postbiotics are being researched [46,47] as safe and effective alternatives to overcome the safety, functionality, and stability limitations of probiotics [48].

This study explored the advantages of postbiotics, focusing on beLP-K produced through heat-killed processing of a Korean-made kimchi-derived strain. This study determined whether heat-killed beLP-K can alleviate muscle atrophy in DEX-treated C2C12 myotubes by downregulating pathways associated with muscle degradation. Specifically, it elucidated the mechanism whereby beLP-K ameliorates sarcopenia.

## 2. Results

### 2.1. Effects of beLP-K on the Viability of C2C12 Myoblasts and Myotubes

**Figure 1:**
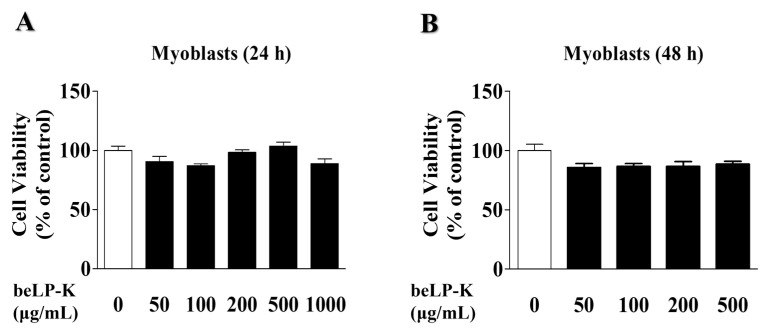
Cytotoxic effects of beLP-K in C2C12 myoblasts. (**A**) C2C12 myoblasts were treated with beLP-K at concentrations of 0, 50, 100, 200, and 500 μg/mL, and cultured for 24 and (**B**) 48 h. Cytotoxicity was assessed using the MTT assay. Results are expressed as cell viability relative to the control group. MTT: 3-(4,5-Dimethylthiazol-2-yl)-2,5-diphenyltetrazolium bromide.

**Figure 2:**
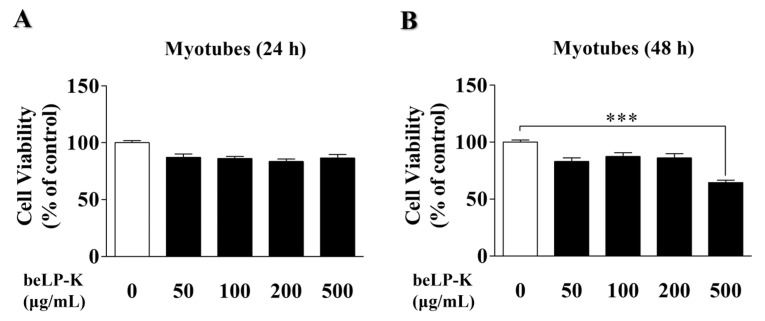
Cytotoxic effects of beLP-K in C2C12 myotubes. (**A**) C2C12 myotubes were treated with beLP-K at concentrations of 50–500 μg/mL and cultured for 24 and (**B**) 48 h. Cytotoxicity was assessed using the MTT assay. Results are presented as cell viability relative to the control group. Data are presented as the mean ± SEM (*n* = 4); *** *p* < 0.001 compared to the control group. MTT: 3-(4,5-Dimethylthiazol-2-yl)-2,5-diphenyltetrazolium bromide.

To investigate beLP-K cytotoxicity, its effects on C2C12 myoblast and differentiated C2C12 myotube viability were evaluated using the 3-(4,5-Dimethylthiazol-2-yl)-2,5-diphenyltetrazolium bromide (MTT) assay. C2C12 myoblasts were treated with various concentrations of beLP-K (50, 100, 200, and 500 μg/mL) and cultured for 24 and 48 h. beLP-K did not exhibit significant cytotoxic effects even at its highest concentration (500 μg/mL) over 24 and 48 h of cell culturing (Figure 1A,B). Therefore, under the same concentration conditions, C2C12 myoblasts were induced to differentiate into myotubes by replacing the medium at 2 d intervals for 4 d, and beLP-K was diluted using the differentiation medium (DM; Dulbecco’s Modified Eagle Medium + 2% horse serum) to investigate its cytotoxicity on C2C12 myotubes. Cell viability was unaffected after incubation for 24 h at various beLP-K concentrations (50, 100, 200, and 500 μg/mL; Figure 2A). However, after incubation to 48 h at the same beLP-K concentration, a sharp decrease was observed in cell viability at 500 μg/mL to <70% (*p* < 0.001; Figure 2B). A cell viability below 70% is typically considered indicative of significant cytotoxicity [49]. Thus, the maximum beLP-K concentration was selected to be 200 μg/mL, and this was the highest concentration with no statistically significant reduction in viability.

### 2.2. Evaluation of the Protective Efficacy of beLP-K in DEX-Induced C2C12 Myotubes

**Figure 3:**
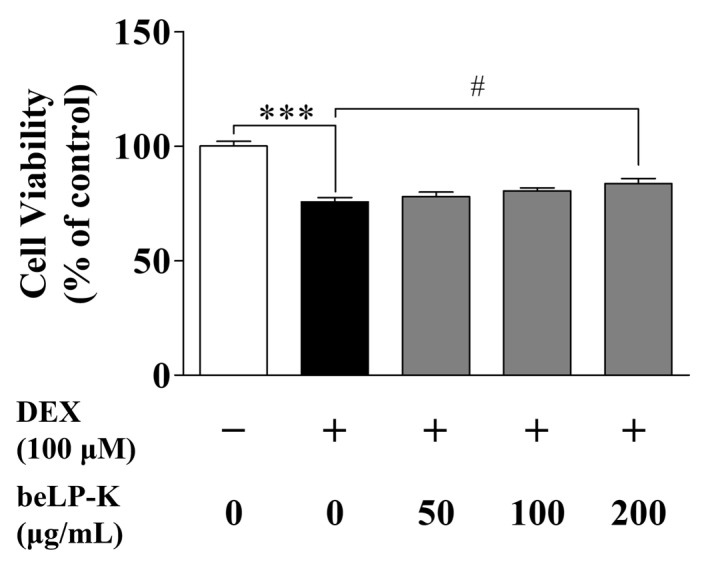
Effects of beLP-K on the viability of DEX-induced cell damage in C2C12 myotubes. Cell viability was assessed using MTT assay. Data are presented as the mean ± SEM (*n* = 4); *** *p* < 0.001 compared to the control group. # *p* < 0.05 compared to the DEX treatment group. DEX: dexamethasone, MTT: 3-(4,5-Dimethylthiazol-2-yl)-2,5-diphenyltetrazolium bromide.

Cell viability was evaluated using an MTT assay. To evaluate viability in DEX-treated C2C12 myoblasts, the myoblasts were seeded onto 24-well plates. Then, at 80–90% confluence, the medium was changed to DM to induce differentiation for 4 days, with the medium changed every 2 days. Subsequently, the cells were cultured with beLP-K at 50–200 μg/mL for 48 h; then, after removing beLP-K, the cells were treated with 100 μM DEX for 24 h to assess cell damage. In this study, the concentration of DEX was selected based on previous studies that applied 100 μM in sarcopenia-like models using C2C12 cells [50,51]. In the control group (without beLP-K, DEX), cell viability was 100 ± 4.26%. In the DEX treatment group, cell viability decreased to 75.73 ± 3.58%. However, in the beLP-K treatment group, cell viability increased in a concentration-dependent manner to 78.04 ± 3.80% (50 μg/mL), 80.56 ± 2.46% (100 μg/mL), and 83.69 ± 4.27% (200 μg/mL) (Figure 3).

### 2.3. Morphometric Analysis of the Efficacy of beLP-K Against DEX-Induced Cell Damage in C2C12 Myotubes

**Figure 4:**
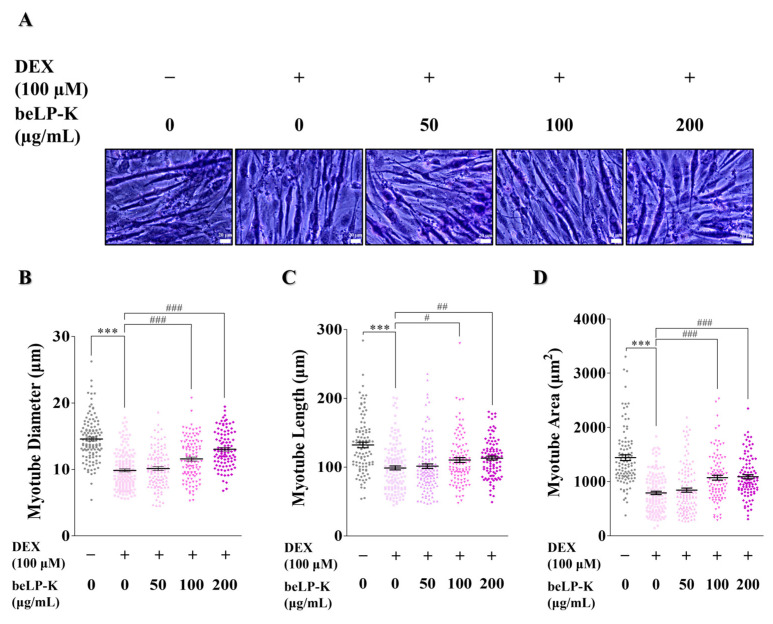
Effects of beLP-K against DEX-induced cell damage in C2C12 myotubes and morphological analysis. (**A**) Depiction of stained C2C12 myotubes after 24 h of treatment with 100 μM DEX to induce cell damage (scale bar: 20 μm). (**B**–**D**) Comparative analysis of myotube diameter, length, and area between the beLP-K treatment groups following treatment with DEX. All images were randomly selected from three independent experiments and analyzed using ImageJ software (version 1.8.0.170). Data are presented as the mean ± SEM (*n* = 15 per group); *** *p* < 0.001 compared with the control group; # *p* < 0.05, ## *p* < 0.01 and ### *p* < 0.001 compared with the DEX treatment group. DEX: dexamethasone.

Morphological analysis was conducted using May-Grünwald-Giemsa staining to assess the protective effects of beLP-K against DEX-induced sarcopenia. beLP-K exhibited effective protective effects in the beLP-K treatment group, following differentiated C2C12 myotube treatment with 100 μΜ DEX (Figure 4A). Compared to the control group, the myotube diameter decreased in the DEX treatment group by 32.3%, indicating that DEX induced myotube damage. However, based on the diameter in the DEX treatment group, the beLP-K treatment groups exhibited damage reduction rates of 5.62% (50 μg/mL), 36.3% (100 μg/mL), and 67.5% (200 μg/mL) in a concentration-dependent manner (Figure 4B). In addition, the myotube length in the DEX treatment group decreased by 25.2% compared to the control group. In contrast, the beLP-K treatment groups exhibited damage reduction rates of 7.97% (50 μg/mL), 34.5% (100 μg/mL), and 42.7% (200 μg/mL) compared to the DEX treatment group (Figure 4C). Furthermore, compared to the control group, the myotube area in the DEX treatment group decreased by 45.1%. However, compared to the DEX treatment group, the beLP-K treatment groups exhibited damage reduction rates of 7.82% (50 μg/mL), 43.2% (100 μg/mL), and 45.7% (200 μg/mL), indicating the protective effects of beLP-K against sarcopenia-like damage (Figure 4D).

### 2.4. Effects of beLP-K on E3 Ubiquitin Proteasome Pathway Regulation in DEX-Induced C2C12 Myotubes

**Figure 5:**
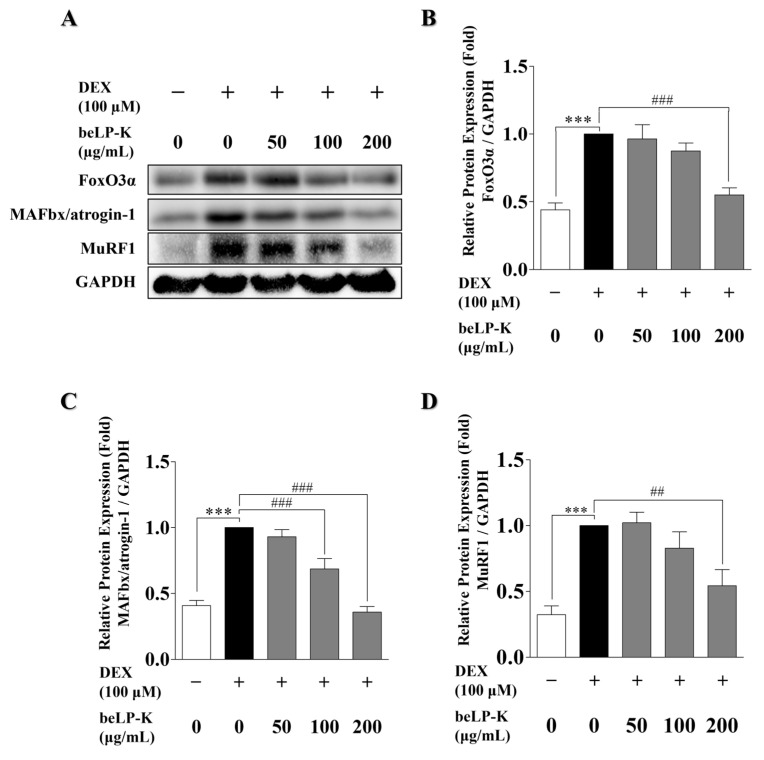
Effects of beLP-K-mediated E3-ubiquitin proteasome pathway inhibition on DEX-induced damage in C2C12 myotubes. (**A**) Western blotting of FoxO3α, MAFbx/atrogin-1, MuRF1, and GAPDH. (**B**) Quantitative analysis of FoxO3α and GAPDH expression (*n* = 4). (**C**) Quantitative analysis of MAFbx/atrogin-1 and GAPDH expression (*n* = 7). (**D**) Quantitative analysis of MuRF1 and GAPDH expression (*n* = 5). GAPDH expression remained stable across all groups and was used to normalize the expression levels of each protein. Results are expressed as multiple changes compared to the DEX treatment group, which was set to 1. Data are presented as the mean ± SEM; *** *p* < 0.001 compared to the control group. ## *p* < 0.01 and ### *p* < 0.001 compared to the DEX treatment group. DEX: dexamethasone, FoxO3α: forkhead box o3 α, MAFbx: muscle atrophy f-box, MuRF1: muscle ring finger protein-1, and GAPDH: glyceraldehyde 3-phosphate dehydrogenase.

To elucidate the mechanisms underlying the inhibitory effects of beLP-K against DEX-induced sarcopenia in C2C12 myotubes, we evaluated protein expression levels through Western blotting. Specifically, we evaluated the expressions of MAFbx/atrogin-1 and MuRF1, key E3-ubiquitin ligases involved in the regulation of muscle protein degradation and synthesis, as well as expression of FoxO3α, a transcription factor that modulates these ligases. Compared to the control group, DEX treatment upregulated the protein expression levels of FoxO3α by 56%, MAFbx/atrogin-1 by 59.2%, and MuRF1 by 67.7% in C2C12 myotubes. Conversely, compared with the DEX treatment group, beLP-K treatment at the highest concentration (200 μg/mL) reduced the expression levels of FoxO3α by 44.9%, MAFbx/atrogin-1 by 64.2%, and MuRF1 by 45.7%, with each approaching the values observed in the control group (Figure 5). These findings suggest that beLP-K modulates the expression of FoxO3α under DEX-induced cellular damage, which is potentially associated with decreased expression of the E3-ubiquitin ligases MAFbx/atrogin-1 and MuRF1, thereby mitigating muscle proteolysis and alleviating GC-induced sarcopenia.

### 2.5. beLP-K Prevents Body Weight and Muscle Volume in DEX-Induced Sarcopenia Rat Model

**Figure 6:**
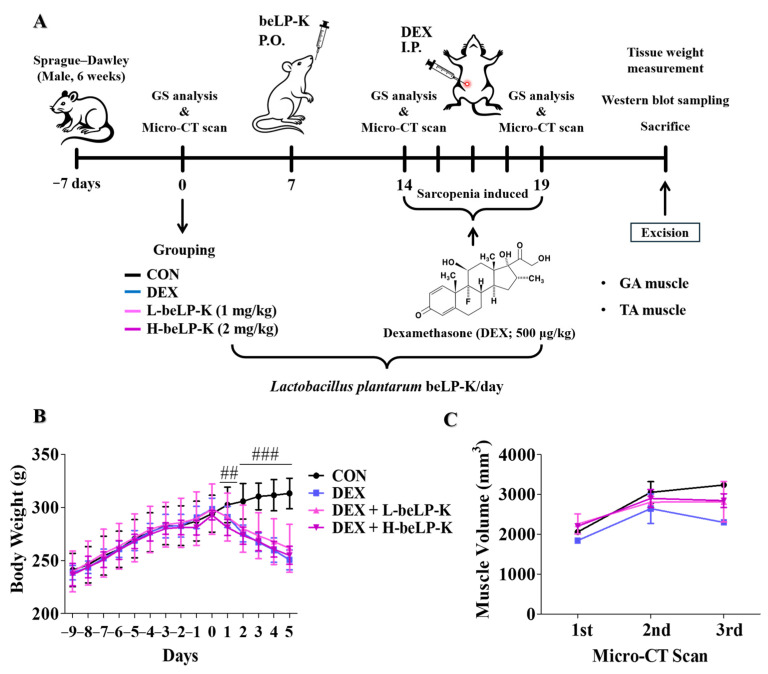
Effects of beLP-K on DEX-induced sarcopenia in SD rats. (**A**) Schematic for drug administration and experimentation on male SD rats. (**B**) Alterations in the body weights of the experimental animals were monitored following beLP-K and DEX administration. (**C**) Changes in muscle volume were assessed thrice through micro-CT scanning. Muscle volume was measured using CT Analyzer software (v1.11). Data are expressed as the mean ± SEM (*n* = 6). Statistical significance was determined using two-way ANOVA; ## *p* < 0.01 and ### *p* < 0.001 compared with the DEX treatment group. DEX: dexamethasone, SD: Sprague–Dawley, CT: computed tomography, GA, gastrocnemius, TA, tibialis anterior, GS: grip strength, L-beLP-K: low-dose beLP-K, H-beLP-K: high-dose beLP-K, I.P.: intraperitoneal, and P.O., per os.

The protective efficacy of beLP-K against DEX-mediated sarcopenia-induced muscle and weight loss was investigated using rat models. We conducted in vivo experiments after establishing a sarcopenia model by administrating beLP-K and DEX to 3-weeks-old male Sprague–Dawley (SD) rats. After an acclimatization period of 1 week, the experimental animals were divided into four groups, i.e., the CON, DEX, low-dose beLP-K, and high-dose beLP-K groups. Group allocation was performed using a random number table without considering variables such as body weight, food intake, or water intake. It was conducted as a blind experiment. Rats in the low-dose beLP-K group were orally administered 1 mg/kg beLP-K diluted in distilled water (DW) for 2 weeks, whereas rats in the high-dose beLP-K group were administered 2 mg/kg beLP-K for 2 weeks. During the same period, rats in the CON and DEX groups were orally administered equal amounts of DW. Subsequently, based on previous studies [52], DEX was administered to rats in the DEX, low-dose beLP-K, and high-dose beLP-K groups at a dose of 500 μg/kg for 5 days, with rats in the CON group administered the same amount of physiological saline for 5 days; then, the rats were sacrificed and analyzed (Figure 6A). The difference in body weight between rats in the different groups was evaluated following an adaptation period. Compared to that in the group, average body weights in DEX-treated rats decreased. However, in rats treated with beLP-K at doses of 1 and 2 mg/kg, weight loss was negligible (Figure 6B). Furthermore, to evaluate the protective effects of beLP-K on muscle mass in DEX-treated rats, a micro-computed tomography (CT) scan was performed thrice before beLP-K administration, after 2 weeks of administration, and before sacrifice to assess changes in muscle mass. Muscle volume was quantified using CT analyzer software (v1.11), with regions of interest designated to include the gastrocnemius and tibialis anterior muscles of the hind limbs. A decrease in muscle volume was observed in the DEX treatment group compared to that in the CON group. However, in the beLP-K treatment group, beLP-K exerted a significant protective effect against this decrease in muscle volume (Figure 6C).

### 2.6. beLP-K Alleviated DEX-Induced Functional Decline and Muscle Loss in SD Rats

**Figure 7:**
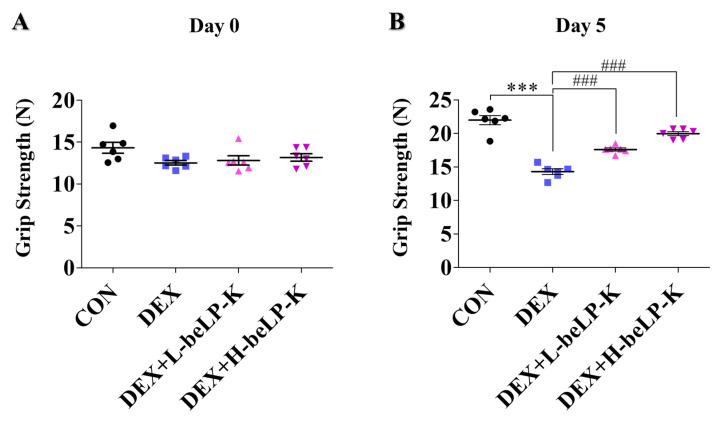
Effects of beLP-K on functional decline in DEX-induced sarcopenia model SD rats. (**A**) Alterations in rat GS prior to DEX administration. (**B**) Alterations in rat GS prior to sacrifice, following DEX administration. GS changes were evaluated and documented 5 d pre- and post-DEX administration, subsequent to oral treatment with beLP-K. GS for each group was assessed thrice per rat. Data are expressed as the mean ± SEM (*n* = 6 per group); *** *p* < 0.001 compared to the CON group. ### *p* < 0.001 compared to the DEX treatment group. DEX: dexamethasone, SD: Sprague–Dawley, DEX: dexamethasone, GS: grip strength, CON: control, L-beLP-K: low-dose beLP-K, and H-beLP-K: high-dose beLP-K.

**Figure 8:**
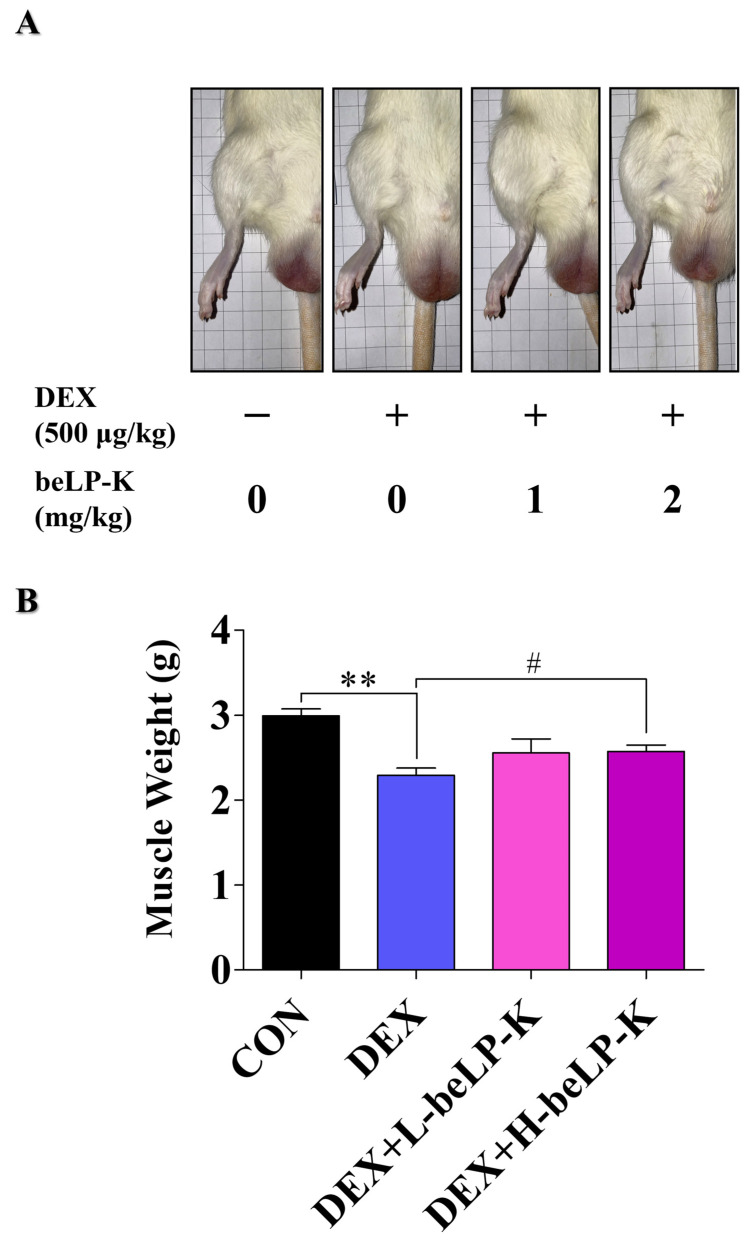
Effects of beLP-K on total tibial muscle weight in DEX-induced sarcopenia model SD rats. (**A**) Comparative depiction of tibial muscles in each group post-sacrifice. (**B**) Comparative analysis of the weights of the extracted tibial muscles. DEX-induced sarcopenia induced significantly lower muscle weight in the beLP-K treatment group than in the DEX treatment group. Data are expressed as the mean ± SEM (*n* = 6 per group); ** *p* < 0.01 compared to the CON group. # *p* < 0.05 compared to the DEX treatment group. DEX: dexamethasone, SD: Sprague–Dawley, DEX: dexamethasone, GS: grip strength, CON: control, L-beLP-K: low-dose beLP-K, and H-beLP-K: high-dose beLP-K.

**Figure 9:**
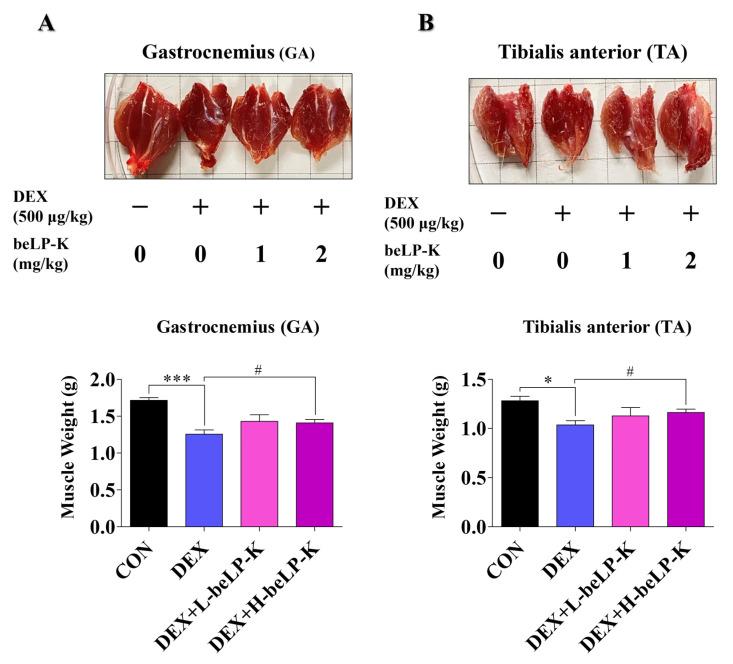
Effects of beLP-K on GA and TA muscle weight in DEX-induced sarcopenia model SD rats. Rats in each experimental group were administered beLP-K for 2 weeks, then administered DEX for 5 d. Muscle tissue form and weight changes were determined for the (**A**) GA and (**B**) TA muscles after the sacrifice of each experimental animal. Data are presented as the mean ± SEM (*n* = 6 per group); * *p* < 0.05 and *** *p* < 0.001 compared to the CON group. # *p* < 0.05 compared to the DEX treatment group. DEX: dexamethasone, SD: Sprague–Dawley, DEX: dexamethasone, GS: grip strength, CON: control, L-beLP-K: low-dose beLP-K, and H-beLP-K: high-dose beLP-K.

Grip strength (GS) was measured to compare muscle strength between the CON group and DEX groups, wherein muscle loss was observed following treatment with DEX. GS was assessed using a grip strength meter, and each animal was tested three times as a blind experiment. After 2 weeks of oral beLP-K administration, no significant difference in GS was observed between the groups before sarcopenia induction (Figure 7A). After sarcopenia induction, measurements were performed 24 h after the final DEX administration to assess the effects of the treatment. Muscle strength significantly decreased in DEX treatment groups compared to that in the CON group. However, in the beLP-K treatment group, muscle strength significantly increased, and beLP-K exerted a protective effect on muscle strength (Figure 7B). To verify the correlation between muscle mass and strength under this muscle strength protective effect, rat leg muscles were analyzed before extraction and after sacrifice (Figure 8A). Subsequently, two muscles (GA, gastrocnemius; TA, tibialis anterior muscles) in the tibial region were excised from all experimental rats, and their sizes and weights were quantitatively compared. Total muscle weight significantly decreased in the DEX treatment group, whereas it significantly increased in the beLP-K treatment group (Figure 8B). Specifically, the size and weight of the GA muscle, a major lower-limb muscle that provides leg support and contributes to locomotion, were significantly lower in the DEX treatment group than in the CON group. However, in groups wherein beLP-K was administered at doses of 1 and 2 mg/kg, the DEX-induced decrease in muscle mass was suppressed (Figure 9A). The TA muscle, a large leg muscle that is a key contributor to ankle dorsiflexion and essential for stable gait, experienced significant loss in the DEX treatment group, whereas muscle size and weight significantly increased in the beLP-K treatment group (Figure 9B) [53]. Overall, beLP-K exerted protective effects against DEX-mediated muscle loss.

### 2.7. beLP-K Decreases E3-Ubiquitin Ligase Expression in DEX-Treated SD Rats

**Figure 10:**
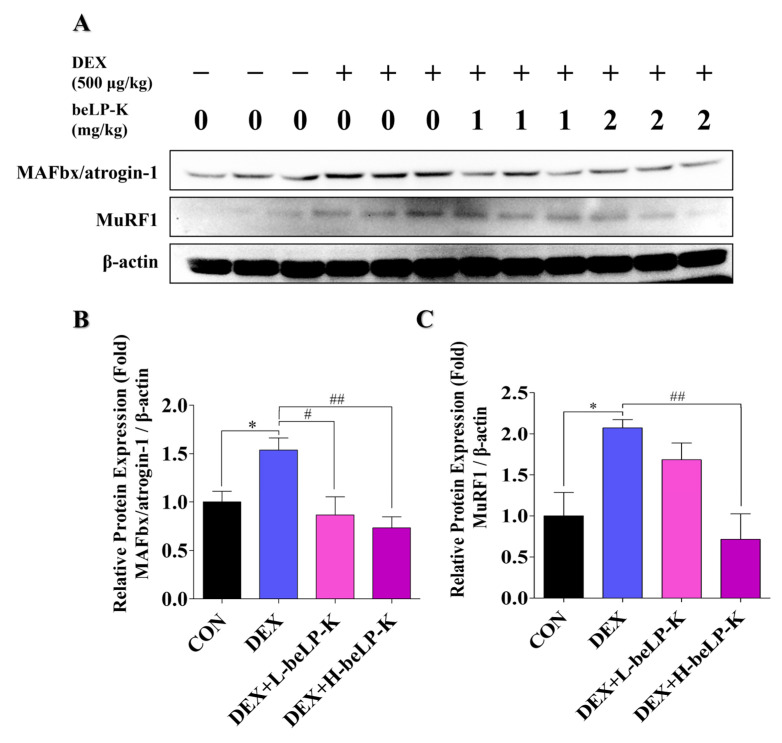
beLP-K attenuates sarcopenia by suppressing the expression of muscle degradation-related proteins in the DEX-induced SD rat model. (**A**) Representative Western blot analysis of MAFbx/atrogin-1, MuRF1, and β-actin in GA. (**B**) Comparison of MAFbx/atrogin-1 protein expression in the GA group. (**C**) Comparison of MuRF1 protein expression levels in each group. Protein expression levels were normalized to β-actin for quantification. Data are presented as the mean ± SEM (*n* = 3); * *p* < 0.05, compared to the CON group. # *p* < 0.05 and ## *p* < 0.01 compared to the DEX group. DEX: dexamethasone, SD: Sprague–Dawley, DEX: dexamethasone, GA: gastrocnemius, MAFbx: muscle atrophy f-box, MuRF1: muscle ring finger protein 1. CON: control, L-beLP-K: low-dose beLP-K, and H-beLP-K: high-dose beLP-K.

To investigate the mechanisms underlying the sarcopenia-induced changes in muscle strength and weight, the expression levels of E3-ubiquitin ligase were determined in GA muscle tissues. Proteins extracted from the GA muscle were analyzed by Western blotting, and the effect of beLP-K on DEX-induced MAFbx/atrogin-1 and MuRF1 expression in each group was investigated (Figure 10A). After treatment with 500 μg/kg of DEX for 5 days, the expression of proteins involved in the E3-ubiquitin proteasome pathway increased in treated rats compared to their expression in CON rats. In rats orally administered beLP-K at 1 and 2 mg/kg, the expression levels of MAFbx/atrogin-1, a protein that mediates muscle degradation through the E3-ubiquitin proteasome pathway during DEX-induced sarcopenia, significantly decreased (Figure 10B). In addition, oral beLP-K administration significantly decreased the expression of MuRF1, another muscle loss-related marker protein, in DEX-treated rats (Figure 10C). These results suggest that beLP-K inhibits the activation of the DEX-induced ubiquitin-proteasome pathway, leading to reduced muscle degradation.

## 3. Discussion

With the significant increase in life expectancy worldwide, the number of elderly people is gradually increasing. This trend has increased the medical expenditure economic burden in several countries [54,55] Sarcopenia incidence in the geriatric population is particularly high; thus, there is a need for the development of new treatment methods for sarcopenia [56]. In most studies, models established using DEX, a synthetic GC analog, have been widely used to treat anti-inflammatory and metabolic diseases [57,58], with continuous administration required in patients with Cushing’s syndrome and congenital adrenal hyperplasia [59]. However, high-dose and/or long-term DEX administration induces several side effects, including sarcopenia. Thus, in several in vitro studies, DEX was used to induce sarcopenia in muscle-related research models as it significantly increases the expression of MAFbx/atrogin-1 and MuRF1, key regulators of UPS activity, in C2C12 myotubes [60,61,62]. A recent study reported that *L. plantarum* HY7715 and *L. rhamnosus* JY02, kimchi-derived strains, can be used as lactobacillus food materials to alleviate DEX-induced muscle loss [63,64]. In this study, beLP-K postbiotics exhibited inhibitory effects against sarcopenia by protecting muscle mass and strength, confirming their therapeutic potential.

Based on the findings of previous studies, we established a DEX-induced sarcopenia model by administering DEX to C2C12 myotubes (in vitro model) and SD rats (in vivo model) [50,51,65,66,67]. We found that in C2C12 myotubes, beLP-K did not decrease cell viability at concentrations up to 200 μg/mL upon culturing for 24 or 48 h (Figure 2). Based on the findings of previous studies wherein muscle loss was induced by treating C2C12 myotubes with DEX, we selected an appropriate DEX concentration for this study. Treatment with 100 μM DEX significantly induced C2C12 myotube damage. However, pre-treatment with beLP-K exerted preventive effects against DEX-induced C2C12 myotube damage (Figure 3). beLP-K protected C2C12 myotubes against DEX-induced muscle loss. Although treatment with DEX decreased muscle size and shape, beLP-K exerted a significant protective effect on muscle size as it comprehensively suppressed the decrease in muscle diameter, length, and area (Figure 4). DEX induced sarcopenia in relation to the expression of MAFbx/atrogin-1 and MuRF1, muscle-specific E3-ubiquitin ligases that inhibit muscle proteins degradation and synthesis by the UPS [38,68,69]. MAFbx/atrogin-1 and MuRF1 have been identified as representative biomarkers in muscle-related studies, as they are involved in muscle weakness and reduction; this is because they mediate protein ubiquitination to promote degradation [70,71]. FoxO3α is an important transcriptional factor that regulates the expression of UPS proteins as well as that of MAFbx/atrogin-1 and MuRF1.

We determined the expression levels of FoxO3α, MAFbx/atrogin-1, and MuRF1, which mediate E3-ubiquitin ligase gene expression and muscle protein degradation through the UPS. Consequently, beLP-K significantly downregulated FoxO3α expression (Figure 5), suggesting that MAFbx/atrogin-1 and MuRF1 expression can be downregulated by downregulating the UPS through decreasing the intracranial migration of FoxO3α, preventing it from performing its function as a transcription factor. Collectively, beLP-K effectively inhibited DEX-induced sarcopenia in C2C12 myotubes. Therefore, we evaluated the in vivo protective effects of beLP-K against the decrease in muscle strength and mass in DEX-treated SD rats. During the experimental period, the rats were weighed daily to note changes in their body weight. Compared to rats in the CON group, rats in the DEX treatment group exhibited weight loss, whereas those in the beLP-K treatment group exhibited weight gain. In addition, we observed that muscle volume in the beLP-K treatment group was greater than that in the DEX treatment group; thus, the DEX-induced decrease in muscle mass was suppressed. Furthermore, a significant concentration-dependent increase in GS was observed in the beLP-K treatment group compared to that observed in the DEX group, confirming that beLP-K improves muscle strength.

**Figure 11:**
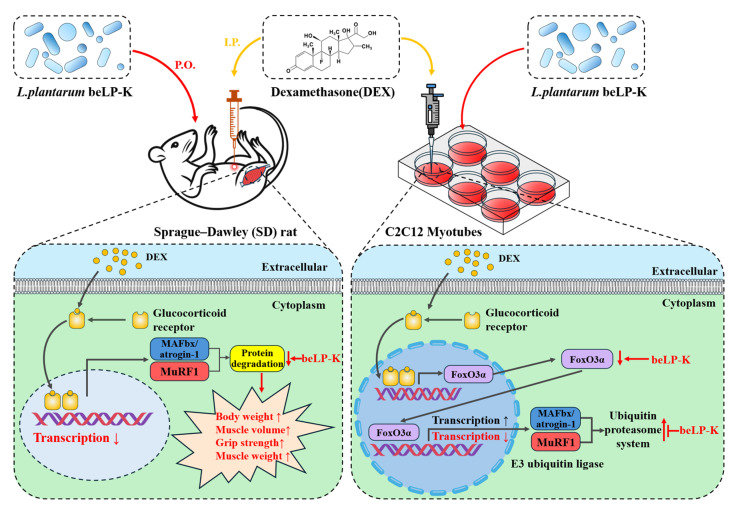
Schematic diagram of the mechanisms of beLP-K in modulating sarcopenia in DEX-induced C2C12 myotubes and SD rats. Red arrows indicate the effects of beLP-K; yellow arrows indicate DEX administration in vivo and in vitro; and gray arrows represent signaling pathways downstream of glucocorticoid receptor activation. DEX: dexamethasone, I.P.: intraperitoneal, P.O.: per os, SD: Sprague–Dawley, FoxO3α: forkhead box o3 α, MAFbx: muscle atrophy f-box, and MuRF1: muscle ring finger protein-1.

Among the leg muscles, GA and TA are composed of a mixture of types I and II fibers, with >50% being type II fibers [72,73,74]. Previous studies have shown a possible difference in response to GCs, such as DEX, between types I and II fibers, and sarcopenia is mostly associated with a decrease in type II fibers [75,76,77,78,79]. Thus, compared to the CON group, for the GA and TA muscles, which have extensive type II fiber distribution, overall muscle weight decreased in the DEX treatment group, whereas muscle weight gradually increased in the beLP-K treatment group. Collectively, beLP-K suppressed the loss of type II fibers induced by DEX and alleviated the reduction in muscle mass. The UPS constitutes the most important signaling pathway in sarcopenia pathophysiology. Several studies have shown that muscle loss is caused by UPS-mediated MAFbx/atrogin-1 and MuRF1 expression under FoxO3α transcriptional regulation [80,81]. In the type II fiber muscles extracted in this study, beLP-K inhibited the expressions of MAFbx/atrogin-1 and MuRF1, muscle-degrading proteins that act as E3-ubiquitin ligases. Collectively, this study demonstrates that beLP-K regulates UPS-related molecular markers involved in muscle protein degradation and protects muscle strength and mass. However, to clearly elucidate the molecular mechanisms underlying these effects, further studies are required to isolate and characterize the active components of beLP-K and to compare its efficacy with that of known antioxidants. Nevertheless, these findings suggest that beLP-K suppresses muscle protein degradation and mitigates the loss of muscle strength and mass, providing fundamental data for future research on sarcopenia (Figure 11).

## 4. Materials and Methods

### 4.1. Preparation of Heat-Killed L. plantarum Postbiotic beLP-K

The beLP-K used in this study was supplied by bereum Co., Ltd. (Wonju, Republic of Korea). The postbiotics were prepared using heat-killed *L. plantarum* strains isolated from kimchi, a traditional Korean fermented vegetable dish, in 2021, consisting of inactivated components of *L. plantarum* with potential biological activity. The concentration of the inactivated bacterial strain was 3.0 × 10^12^ cells per gram.

### 4.2. Cell Experiment Design Using C2C12 Myoblasts

Mouse-derived skeletal muscle cell line C2C12 myoblasts were used in this study. The cells were cultured in Dulbecco’s Modified Eagle Medium (Sigma-Aldrich, St. Louis, MO, USA) containing 1% (*v*/*v*) penicillin/streptomycin (100×; Sigma-Aldrich) and 10% (*v*/*v*) fetal bovine serum (Access Biologics LLC., Vista, CA, USA) at 37 °C. Next, the cells were seeded in six-well culture plates at a density of 2.0 × 10^5^ cells/well. When cell confluence reached 80–90%, the medium was replaced with DM, a differentiation medium containing 2% (*v*/*v*) horse serum (Gibco, Thermo Fisher Scientific Inc., Waltham, MA, USA), and 1% penicillin/streptomycin to induce myotube differentiation; subsequently, the medium was replaced at 2 days intervals. After differentiation, the cells were pre-treated with beLP-K (50, 100, and 200 μg/mL) for 48 h to evaluate its preventive potential. Subsequently, the cells were exposed to DEX (100 μM) for 24 h to induce cell damage.

### 4.3. Cell Viability Assay

beLP-K cytotoxicity was assessed using the MTT assay. C2C12 myoblasts were seeded at 5.0 × 10^4^ cells/well and incubated for 24 h at 37 °C in 5% CO_2_. To evaluate beLP-K cytotoxicity in myotubes and its protective efficacy against DEX-induced damage, C2C12 myoblasts were seeded at 1.0 × 10^5^ cells/mL and cultured to 80–90% confluence. The medium was replaced with DM and subsequently replaced every 2 days for 4 days. Then, beLP-K was added at various concentrations (0, 50, 100, 200, and 500 μg/mL) and incubated for 24 and 48 h. Next, the cells were treated with DEX at 100 μM for 24 h to cellular damage associated with sarcopenia. Thereafter, 10 µL of MTT solution (5 mg/mL) per 100 µL of medium and incubated for 2 h. Subsequently, the medium was removed, and the formed purple formazan crystals were immediately dissolved in dimethyl sulfoxide. Optical density was measured at 595 nm using an FLx800 microplate reader (BioTek Instruments Inc., Winooski, VT, USA), with untreated cells used the negative control and cells treated with 100 µM DEX alone as the positive control.

### 4.4. Morphological Assessment

Morphological analysis of DEX-induced C2C12 myotubes was performed using May–Grünwald–Giemsa staining, based on previous studies [82,83]. DEX-induced C2C12 myotubes were washed three times with phosphate-buffered saline (PBS; pH 7.4) and fixed with cold absolute methanol at −20 °C for 5 min. After fixation, the cells were washed with PBS and air-dried. And the cells were first stained with a May–Grünwald staining solution (Sigma-Aldrich), which was diluted 1:3 in PBS (pH 6.8), for 5 min. Subsequently, cells were washed in PBS, and Giemsa staining working solution (Sigma-Aldrich) was diluted 1:10 in DW for 10 min. After staining, the cells were gently washed three times with DW to remove residual staining solution. After staining, the cells were gently washed three times with DW to remove residual staining solution. The stained cells were visualized at 200× magnification using a Nikon inverted microscope (Nikon Corp., Tokyo, Japan), and the diameter, length, and area of the myotubes were measured using ImageJ software (National Institutes of Health, Bethesda, MD, USA). The measurement of diameter, length, and area of the myotubes was performed using five randomly selected images per group from three independent experiments (*n* = 15 per group), with the number of myotubes per image varying depending on cell density within the field.

### 4.5. Western Blot Analysis and Antibodies

Protein expression levels in cells treated with beLP-K to induce protective effects against sarcopenia were assessed by Western blotting. Protein extracts were prepared by lysis using the PRO-PREP™ protein extraction solution (iNtRON Biotechnology Inc., Seongnam, Republic of Korea). Protein concentration was determined using the Bradford assay (Bio-Rad, Hercules, CA, USA). Protein samples were separated according to molecular weight by sodium dodecyl sulfate–polyacrylamide gel electrophoresis at 100 V for approximately 2 h. After electrophoresis, the gel was transferred to an Immune-Blot polyvinylidene difluoride membrane (Bio-Rad), which was incubated overnight with the indicated primary antibodies at 4 °C. Subsequently, horseradish peroxidase (HRP)-linked anti-rabbit IgG secondary antibodies were added to the membrane and incubated at RT for approximately 1 h. Chemiluminescence-based detection of protein bands was performed by treating the polyvinylidene difluoride membranes with WestGlow™ PICO PLUS (Biomax Inc., Guri, Republic of Korea); protein bands were imaged using the ChemiDoc™ XRS+. imaging system with Image Lab™ Software (version 6.0.1; Bio-Rad). Image analysis was performed using ImageJ software (National Institutes of Health). Anti-FoxO3α (Cat. No. #12829; 1:2500), anti-GAPDH; Cat. No. #5174; 1:5000), anti-β-actin (Cat. No. #4967; 1:5000), and HRP-linked anti-rabbit IgG (Cat. No. #7074; 1:5000) antibodies were purchased from Cell Signaling Technology (Danvers, MA, USA). Anti-MuRF-1 (Cat. No. sc-32920; 1:2500) antibodies were purchased from Santa Cruz Biotechnology (Santa Cruz, CA, USA). Anti-MAFbx/atrogin-1 (Cat. No. ab168372; 1:2500) antibodies were purchased from Abcam (Cambridge, MA, USA). Detected protein levels were normalized to GAPDH levels in cell lysates and to β-actin levels in tissue samples. The dilution factors of the antibodies used in Western blotting are shown in parentheses next to the catalog numbers.

### 4.6. Animal Experiment Design

Based on findings from our cell-based experiments, animal experiments were conducted on 6-week-old male SD rats (Samtako Co., Osan, Republic of Korea) with an average weight of 180 g. The breeding environment was designed to allow access to standard rodent feed (Purina Co., Ltd., Yangpyeong, Republic of Korea) and DW, and environmental conditions included a temperature of 22 ± 1 °C, humidity of 45 ± 10%, and a 12 h light/dark cycle. After a 7 days adaptation period, the experimental animals were randomly assigned and reared in four groups (*n* = 6 per group): the negative control (CON), positive control (DEX), low-dose beLP-K (1 mg/kg), and high-dose beLP-K (2 mg/kg) groups. Groups were measured and analyzed by blind experiments. beLP-K diluted in DW was orally administered to rats in each group daily at a volume of 400 μL, while rats in the CON and DEX groups were orally administered 400 μL of DW simultaneously. After oral beLP-K administration for 2 weeks, DEX (500 μg/kg) was injected into rats in the DEX and beLP-K groups for 5 d to induce sarcopenia. At the end of the experimental period, the rats were sacrificed via CO_2_ inhalation, and their hind leg muscle tissues were excised for mass and size measurements. All animal experiments were approved by the Yonsei University Mirae Campus Institutional Animal Care and Use Committee (YWCI-202309-021-01) and carried out in accordance with the regulations.

### 4.7. GS Analysis

GS was assessed in all experimental animals to evaluate the efficacy of beLP-K in mitigating the DEX-induced decrease in muscle strength and function. GS was measured using a grip strength meter (Jeongdo Bio & Plant Co., Seoul, Republic of Korea). Each rat was positioned to hold the grip strength meter, and GS was measured by gently holding and applying a consistent backward pull on the tail. Measurements were taken thrice, i.e., before beLP-K administration, 2 weeks after beLP-K administration, and before sacrifice. The GS of each rat was measured and analyzed thrice at each interval.

### 4.8. Statistical Analysis

All experimental data were analyzed using *t*-tests and one-way analysis of variance, followed by Dunnett’s multiple-comparisons test for post hoc analysis. Data are expressed as the mean ± standard error of the mean. Statistical significance was set at *p* < 0.05, 0.01, and 0.001.

## 5. Conclusions

The inhibitory effect of beLP-K against sarcopenia is attributable to its ability to counteract the DEX-induced increase in the expression of muscle loss-related proteins both in myotubes and muscle tissues. This effect is mediated through downregulation of the protein expressions of FoxO3α, MAFbx/atrogin-1, and MuRF1, which are integral components of the UPS. These findings suggest that beLP-K significantly reduced the risk of complications associated with muscle-related diseases, such as sarcopenia and muscle atrophy. Furthermore, it holds promises for future applications in the prevention, treatment, and improvement of these conditions, as well as in the development of healthy functional foods.

## Data Availability

The data supporting the findings of this study are available from the corresponding author upon reasonable request.

## Abbreviations

The following abbreviations are used in this manuscript:

beLP-K	*Lactobacillus plantarum* beLP-K
CON	Control
CT	Computed tomography
DEX	Dexamethasone
DM	Differentiation medium
DMEM	Dulbecco’s -Modified Eagle Medium
DW	Distilled water
FTMFs	Fast-twitch muscle fibers
FoxO3α	Forkhead box O3 α
GAPDH	Glyceraldehyde 3-phosphate dehydrogenase
GA	Gastrocnemius
GC	Glucocorticoid
GS	Grip strength
GSM	Grip strength meter
HRP	Horseradish peroxidase
ICD	International classification of disease
MAFbx	Muscle atrophy f-box
MuRF1	Muscle RING-finger protein-1
MTT	3-(4,5-Dimethylthiazol-2-yl)-2,5-diphenyltetrazolium bromide
OD	Optical density
PBS	Phosphate-buffered saline
RT	Room temperature
SD	Sprague–Dawley
STMFs	Slow-twitch muscle fibers
TA	Tibialis anterior
UPS	Ubiquitin–proteasome system
WHO	World Health Organization
